# Tetrahydroxystilbene Glucoside Produces Neuroprotection against 6-OHDA-Induced Dopamine Neurotoxicity

**DOI:** 10.1155/2018/7927568

**Published:** 2018-01-14

**Authors:** Chun Huang, Fengqin Lin, Guoqing Wang, Disheng Lu, Qin Wu, Jie Liu, Jingshan Shi, Feng Zhang

**Affiliations:** Joint International Research Laboratory of Ethnomedicine of Ministry of Education and Key Laboratory of Basic Pharmacology of Ministry of Education, Zunyi Medical University, Zunyi, Guizhou, China

## Abstract

Parkinson's disease (PD) was one of the most common neurodegenerative diseases with a slow and progressive loss of dopamine (DA) neurons in the midbrain substantia nigra (SN). Neuroinflammation was identified to be an important contributor to PD pathogenesis with the hallmark of microglia activation. Tetrahydroxystilbene glucoside (TSG) was the main active component extracted from *Polygonum multiflorum* and held amounts of pharmacological activities including antioxidant, free radical-scavenging, anti-inflammation, and cardioprotective properties. Recent studies demonstrated that TSG exerted neuroprotection from several neurodegenerative disease models. However, the underlying mechanisms were not completely elucidated. In the present study, rat nigral stereotaxic injection of 6-hydroxydopamine- (6-OHDA-) elicited DA neuronal injury was performed to investigate TSG-mediated neuroprotection on DA neurons. In addition, primary rat midbrain neuron-glia cocultures were applied to explore the mechanisms underlying TSG-exerted neuroprotection. Results showed that daily intraperitoneal injection of TSG for 14 consecutive days significantly protected DA neurons from 6-OHDA-induced neurotoxicity and suppressed microglia activation. Similar neuroprotection was shown in primary neuron-glia cocultures. *In vitro* studies further demonstrated that TSG inhibited microglia activation and subsequent release of proinflammatory factors. Moreover, TSG-mediated neuroprotection was closely related with the inactivation of mitogen-activated protein kinase (MAPK) signaling pathway. Together, TSG protects DA neurons from 6-OHDA-induced neurotoxicity via the inhibition of microglia-elicited neuroinflammation. These findings suggest that TSG might hold potential therapeutic effects on PD.

## 1. Introduction

Parkinson's disease (PD) is the second most prevalent neurodegenerative disease characterized by a selective loss of dopamine (DA) neurons in the midbrain substantia nigra (SN) [1, 2]. Although the pathogenesis of PD is not completely elucidated, microglia-mediated neuroinflammation has attracted increasing attention [3]. Microglia are the resident immune cells in the brain, acting as the first line of defense from external stimuli. Once activated by brain damage or immune stimuli, microglia release amounts of proinflammatory factors, such as interleukin 1*β* (IL-1*β*), nitric oxide (NO), and tumor necrosis factor *α* (TNF-*α*). The accumulation of these factors led to the surrounded DA neuronal injury [4]. Taken together, microglia-induced neuroinflammation might be a key event in the degradation of DA neurons and the inhibition of microglia activation could possess a promising therapeutic potential for PD [5].

Tetrahydroxystilbene glucoside (TSG), the main active component derived from *Polygonum multiflorum*, holds a large amount of pharmacological properties including antioxidant, free radical-scavenging, anti-inflammation, and cardioprotective effects [6]. Current evidence indicated that TSG has significant neuroprotective properties on ischemic brain both *in vitro* and *in vivo* [7]. Additionally, TSG could not only improve memory and motor function but also attenuate *α*-synuclein aggregation in the striatum of aged mice [8, 9]. However, the mechanisms through which TSG-exerted neuroprotection are not completely elucidated. In the present study, rat nigral stereotaxic injection of 6-hydroxydopamine- (6-OHDA-) elicited DA neuronal injury was performed to investigate TSG-mediated neuroprotection on DA neurons. Furthermore, primary rat midbrain neuron-glia cocultures were applied to explore the mechanisms underlying TSG-produced neuroprotection.

## 2. Materials and Methods

### 2.1. Reagents

TSG (purity > 99%) was obtained from the National Institute for the Control of Pharmaceutical and Biological Products (Beijing, China). 6-OHDA was bought from Sigma-Aldrich (St. Louis, MO, USA). Anti-CR3 complement receptor (OX-42) and tyrosine hydroxylase (TH) antibodies were bought from Abcam Inc. (Cambridge, MA, USA). Biotinylated secondary antibodies were from Vector Laboratories (Burlingame, CA, USA). Enzyme-linked immunosorbent assay (ELISA) kits were from R&D Systems (Minneapolis, MN, USA). Griess reagent was obtained from Beyotime Biotechnology (Shanghai, China). Mitogen-activated protein kinase (MAPK) pathway antibodies were the products of Cell Signaling Technology (Beverly, MA, USA).

### 2.2. Animals and Treatment

Male Wistar rats (200–250 g) were bought from the Experimental Animal Center in the Third Military Medical University. Animal breeding and housing were strictly performed in the accordance with the Animal Care and Use Guidelines of China. To investigate the neuroprotective actions of TSG on 6-OHDA-elicited neurotoxicity, male rats were randomly divided into control, TSG (50 mg/kg) alone, 6-OHDA group, 6-OHDA + TSG (10 mg/kg), and 6-OHDA + TSG (50 mg/kg) groups. Rats received a single 6-OHDA injection (8 *μ*g) into the midbrain SN on the left side of the brain, followed by the coordinates 5.2 mm posterior to bregma, 2.4 mm lateral to the midline, and 8.0 mm ventral to the surface of the skull [10]. Then, TSG was intraperitoneally injected into rats daily for 14 consecutive days. After the last TSG treatment, rats were allowed to recover for an additional 2 weeks development and then the behavior tests (rotarod test) were applied. Afterwards, animals were sacrificed and the biochemical analysis was performed.

### 2.3. Rotarod Test

Rotarod test was performed to study the muscular coordination. It contained cylindrical arrangement of the thin steel rods with two parts by compartmentalization to permit the detection of two rats at the same time. In the train, the speed was set at 10 cycles/min and the cutoff time was 180 s. Before the start of the test, rats were trained on the rotarod until they remained on the rod no less than the cutoff time [11]. Animals were allowed to keep stationary for a while at 0 rpm. The rotational speed was gradually increased to 10 rpm in 20 s interval till rats fell off the rungs. Animals were detected for two trials per day, and the mean duration time that they remained on the rod was recorded.

### 2.4. High-Performance Liquid Chromatography (HPLC) Coupled with Electrochemical Detection

Rat striatum levels of DA and its metabolite, 3,4-dihydroxyphenylacetic acid (DOPAC), were measured by HPLC coupled with electrochemical detection. Rat striatum tissues were sonicated in the perchloric acid including the internal standard 3,4-dihydroxybenzylamine. The homogenate was centrifuged, and an aliquot of the supernatant was injected into the HPLC. The mobile phase consisted of acetonitrile, tetrahydrofuran, and monochloroacetic acid (pH 3.0) containing ethylenediaminetetraacetic acid sodium octyl sulfate (200 mg/l) and (EDTA, 50 mg/l). The DA and DOPAC levels were detected and expressed in the quality of wet weight of tissue [12].

### 2.5. Immunohistochemical Analysis and Cell Counting in the SN

Rat brains were cut with a horizontal sliding microtome into 35 *μ*m transverse free-floating sections. A total of 36 consecutive brain slices throughout the entire SN were collected, in which every 6th section was performed for the immunocytochemical analysis. DA neuron was recognized by an anti-tyrosine hydroxylase (TH) antibody, and the activated microglia were identified with an anti-OX-42 antibody. Digital images of TH-positive neurons and OX-42-positive microglia in midbrain SN were acquired by an Olympus microscope (Olympus, Tokyo, Japan). The DA neurotoxicity was evaluated by TH-positive neuron counting, and microglia activation-elicited neuroinflammation was detected by densitometry assessment of OX-42-positive microglia.

### 2.6. Primary Rat Midbrain Neuron-Glia Cocultures

Primary rat neuron-glia cocultures were prepared from the midbrain tissues of embryonic day 14 and 15 rats [12]. In brief, the whole brain was aseptically removed and the mesencephalon was dissected. After removing the blood vessels and meninges, the midbrain tissues were dissociated with a mild mechanical trituration. Then, the dissociated cells were seeded at 5 × 10^5^/well in a 24-well plate. The cocultures were maintained in a humidified atmosphere of 5% CO_2_ and 95% air at 37°C with the maintenance medium. The seven-day-old cocultures were applied for drug treatments. At the time of treatment, cultures were composed of 50% astroglia, 10% microglia, and 40% neurons (including 1% DA neurons) [13].

### 2.7. Immunocytochemical Staining

Formaldehyde (4%)-fixed cells were treated with hydrogen peroxide (1%) and then incubated with the blocking solution. Cultures were incubated at 4°C overnight with primary anti-OX-42 (1 : 300) and anti-TH (1 : 300) antibodies followed by the biotinylated secondary antibody and Vectastain ABC reagents. Color was developed with 3,3′-diaminobenzidine. For the visual TH-positive neuron counting, four representative areas per well of the 24-well plate were counted. In each condition, three wells were applied for cell counting.

### 2.8. IL-1*β*, TNF-*α*, and Nitrite Assay

The TNF-*α* and IL-1*β* levels in the supernatant were determined by ELISA kits. The release of NO was analyzed by detecting the accumulated levels of nitrite (an indicator of NO) with Griess reagent in the medium of neuron-glia cocultures.

### 2.9. Western Blot Analysis

For the whole cell lysate extraction, cultures were homogenized in RIPA lysis buffer. The lysates were incubated on ice for 30 min and then centrifuged at 12000 ×g for 15 min. Protein concentrations were measured using BCA assay. Equal amounts of total protein were separated on 4–12% Bis-Tris Nu-PAGE gel and then transferred to PVDF membranes. Subsequently, the membranes were blocked by 5% nonfat milk and then incubated with the following primary antibodies: ionized calcium-binding adapter molecule-1 (Iba-1, 1 : 800), TH (1 : 500), phosphorylated-p38 (p-p38, 1 : 1000), p38 (1 : 1000), phosphorylated-ERK1/2 (p-ERK1/2, 1 : 1000), ERK1/2 (1 : 1000), and *β*-actin (1 : 2000). The membranes were incubated in horseradish peroxidase- (HRP-) conjugated secondary antibodies (1 : 2500). The blots were developed with the enhanced chemiluminescence (ECL) reagent.

### 2.10. Statistical Analysis

Data were presented as mean ± standard error of the mean (SEM). The statistical significance was analyzed by one-way ANOVA with GraphPad Prism software. When ANOVA showed the significant differences, pairwise comparisons between means were accessed by Bonferroni's post hoc 𝑡-test with correction. A value of *p* < 0.05 was considered statistically significant.

## 3. Results

### 3.1. TSG Produces Neuroprotection against 6-OHDA-Elicited DA Neurotoxicity *In Vivo*

**Figure 1:**
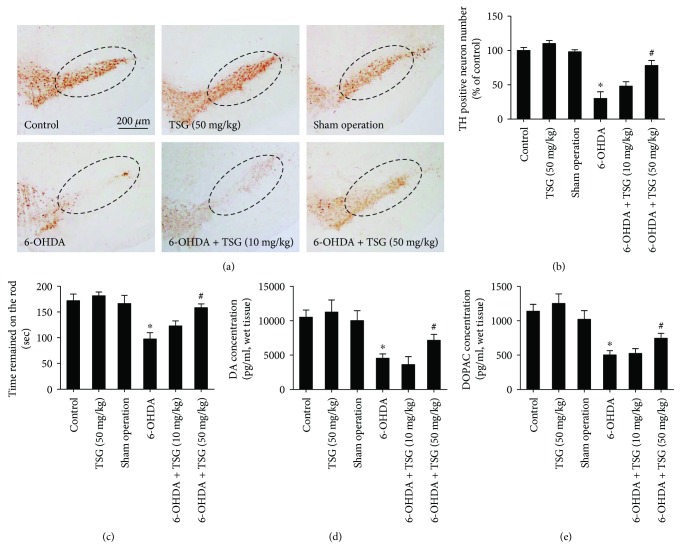
TSG inhibited 6-OHDA-induced DA neuronal loss in the SN *in vivo*. Brain sections were immunostained with an anti-TH antibody (a). The “ellipse” presented the area of SN. Scale bar = 200 *μ*m. The number of TH-positive neurons in the SN was counted (b). Rat behavior changes were analyzed by rotarod test. The time that the rats remained on the rod was recorded (c). The DA (d) and DOPAC (e) levels in rat brain striatum were measured by HPLC coupled with electrochemical detection. Results were expressed as a percentage of the control group and were the mean ± SEM from six rats. ^∗^*p* < 0.05 compared with the control groups; ^#^*p* < 0.05 compared with 6-OHDA-treated group.

After the intraperitoneal injection of TSG for 14 consecutive days, rat brains were collected, sectioned, and processed for quantification of DA neurons by immunohistochemistry staining with anti-TH antibody. As indicated in Figures 1(a) and 1(b), compared with the control group, 6-OHDA significantly reduced the TH-positive neurons in SN, which was ameliorated by TSG treatment. In the rotarod test, TSG attenuated 6-OHDA-induced decrease in the time that the rats remained on the rod shown in Figure 1(c). In addition to DA neuronal number and rat behavior changes, the neuroprotective effects of TSG against 6-OHDA-induced depletion of striatum DA and DOPAC levels were further measured by HPLC coupled with electrochemical detection. As shown in Figures 1(d) and 1(e), in parallel with the reduction of DA neuronal number, a corresponding decrease in DA and DOPAC levels was discerned in 6-OHDA-treated group. However, TSG treatment attenuated 6-OHDA-caused decrease of DA and DOPAC levels in the striatum.

### 3.2. TSG Attenuates 6-OHDA-Induced Microglia Activation *In Vivo*

**Figure 2:**
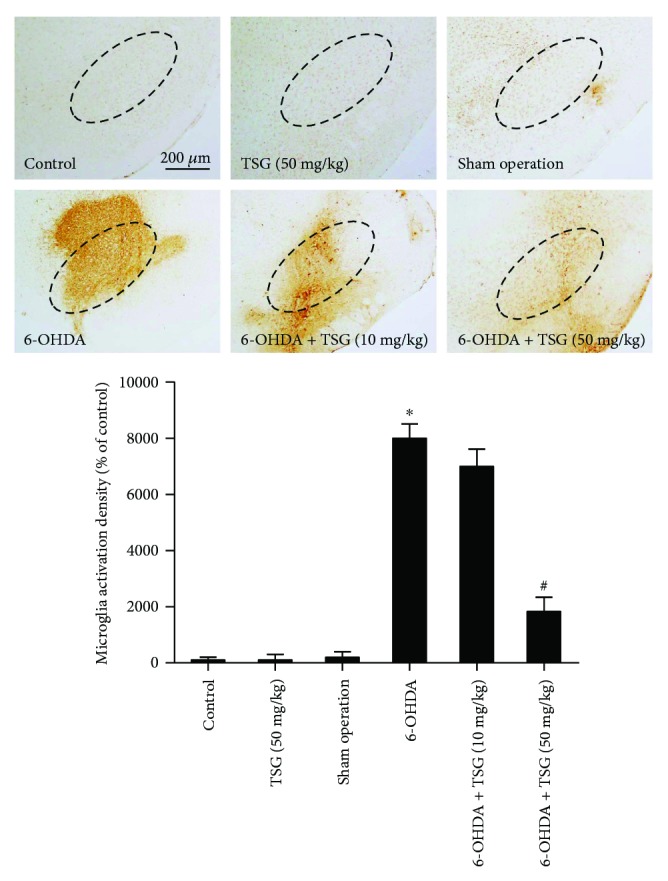
TSG attenuated 6-OHDA-induced microglial activation *in vivo*. Rat brain sections were immunostained by an anti-OX-42 antibody. Densitometry analysis of nigral OX-42-positive microglia was performed with ImageJ software. The “ellipse” presented the area of SN. Scale bar = 200 *μ*m. Quantified results were expressed as a percentage of the control group and were the mean ± SEM from six rats. ^∗^*p* < 0.05 compared with the control groups; ^#^*p* < 0.05 compared with 6-OHDA-treated group.

The brain sections were stained to detect the inhibitory effects of TSG on 6-OHDA-elicited microglia activation. As shown in Figure 2, 6-OHDA induced microglia activation compared with the control group. However, this activated state of microglia was attenuated by TSG treatment.

### 3.3. TSG Protects DA Neurons against 6-OHDA-Induced Neurotoxicity *In Vitro*

**Figure 3:**
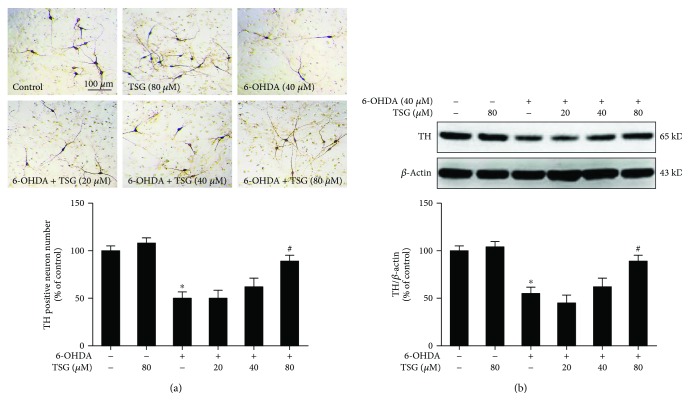
TSG protected DA neurons against 6-OHDA-induced neurotoxicity *in vitro*. 6-OHDA-induced DA neurotoxicity was quantified by TH-positive neuron counting through the immunocytochemical analysis. Representative images of immunostaining from three experiments were shown (a). Scale bar = 100 *μ*m. TH protein expression was determined by western blotting analysis. The ratio of densitometry values of TH with *β*-actin was assessed and normalized to each respective control group (b). Results were expressed as a percentage of the control group and were the mean ± SEM from three independent experiments performed in triplicate. ^∗^*p* < 0.05 compared with the control groups; ^#^*p* < 0.05 compared with 6-OHDA-treated group.

Primary rat midbrain neuron-glia cocultures were treated with TSG (20–80 *μ*M) for 30 min followed by the application of 6-OHDA (40 *μ*M). Seven days later, 6-OHDA-elicited DA damage was quantified by immunocytochemical staining and western blotting assay. As shown in Figure 3(a), TH-positive neuronal counting indicated that 6-OHDA significantly decreased DA neuronal number and TSG ameliorated 6-OHDA-elicited DA neuronal damage. Western blotting analysis also indicated that TSG attenuated 6-OHDA-elicited decrease of TH protein expression (Figure 3(b)). These *in vitro* results were in agreement with the findings from the *in vivo* studies.

### 3.4. TSG Ameliorates 6-OHDA-Elicited Microglia Activation *In Vitro*

**Figure 4:**
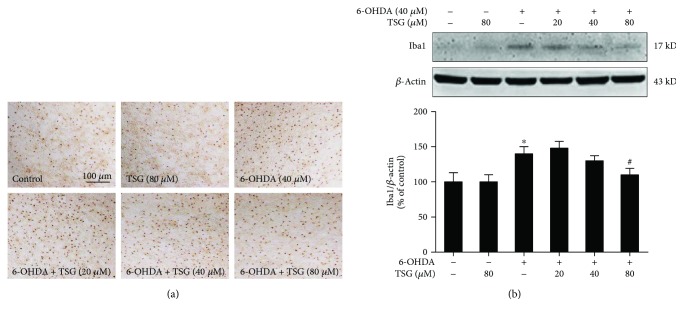
TSG ameliorated 6-OHDA-induced microglial activation *in vitro*. Activated microglia were detected by immunocytochemical staining with an anti-OX-42 antibody. Representative images of immunostaining from three experiments were indicated (a). Scale bar = 100 *μ*m. Iba1 protein expression was determined by western blotting assay. The ratio of densitometry values of Iba1 with *β*-actin was assessed and normalized to each respective control group (b). Results were expressed as a percentage of the control group and were the mean ± SEM from three independent experiments performed in triplicate. ^∗^*p* < 0.05 compared with the control groups; ^#^*p* < 0.05 compared with 6-OHDA-treated group.

Primary midbrain neuron-glia cocultures were pretreated with TSG (20–80 *μ*M) for 30 min and then stimulated by 6-OHDA. Seven days later, the 6-OHDA-induced microglia activation was analyzed. As shown in Figure 4(a), 6-OHDA significantly induced microglia activation. Furthermore, microglia in 6-OHDA-treated cultures presented an enlarged cell body and irregular shapes from the resting small and round cells to the highly activated amoeboid status. However, TSG significantly attenuated 6-OHDA-induced microglia activation. To further investigate the inhibitory properties of TSG on 6-OHDA-induced microglia activation, the whole cell lysis was collected and the effects of TSG on the protein expression of Iba-1 (a marker of microglia used for western blot assay) were detected. As shown in Figure 4(b), 6-OHDA obviously increased Iba-1 protein expression, which was in parallel with TH-positive neuronal loss. TSG reduced 6-OHDA-elevated Iba-1 protein expression.

### 3.5. TSG Decreases the Release of Proinflammatory Factors Induced by 6-OHDA

**Figure 5:**
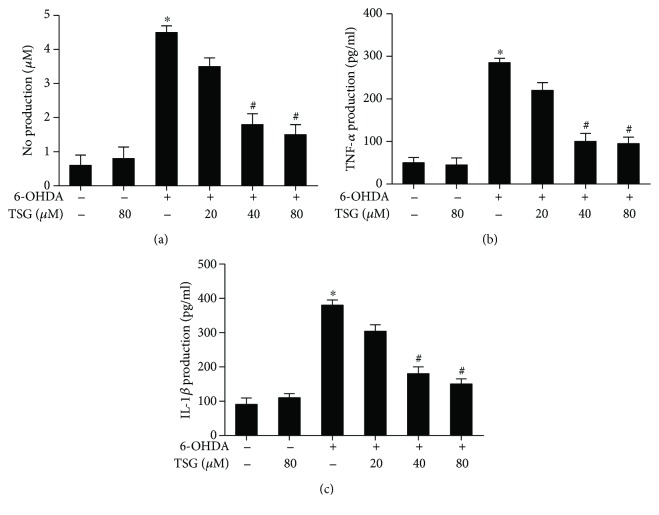
TSG suppressed the release of proinflammatory factors induced by 6-OHDA. The production of NO was determined by Griess reagent (a). The levels of TNF-*α* (b) and IL-1*β* (c) in the culture medium were detected by ELISA. Results were the mean ± SEM from three independent experiments performed in triplicate. ^∗^*p* < 0.05 compared with the control groups; ^#^*p* < 0.05 compared with 6-OHDA-treated group.

The inhibitory actions of TSG on the production of 6-OHDA-induced proinflammatory factors were further explored. Primary rat midbrain neuron-glia cocultures were treated with TSG (20–80 *μ*M) for 30 min followed by 6-OHDA treatment. Twenty-four h later, the production of IL-1*β*, NO, and TNF-*α* in the culture supernatant was detected by ELISA and Griess regent. As shown in Figure 5, the production of IL-1*β*, NO, and TNF-*α* was increased in 6-OHDA-treated cultures. However, compared with 6-OHDA group, TSG significantly suppressed the production of the above proinflammatory factors.

### 3.6. TSG Attenuates 6-OHDA-Induced MAPK Pathway Activation

**Figure 6:**
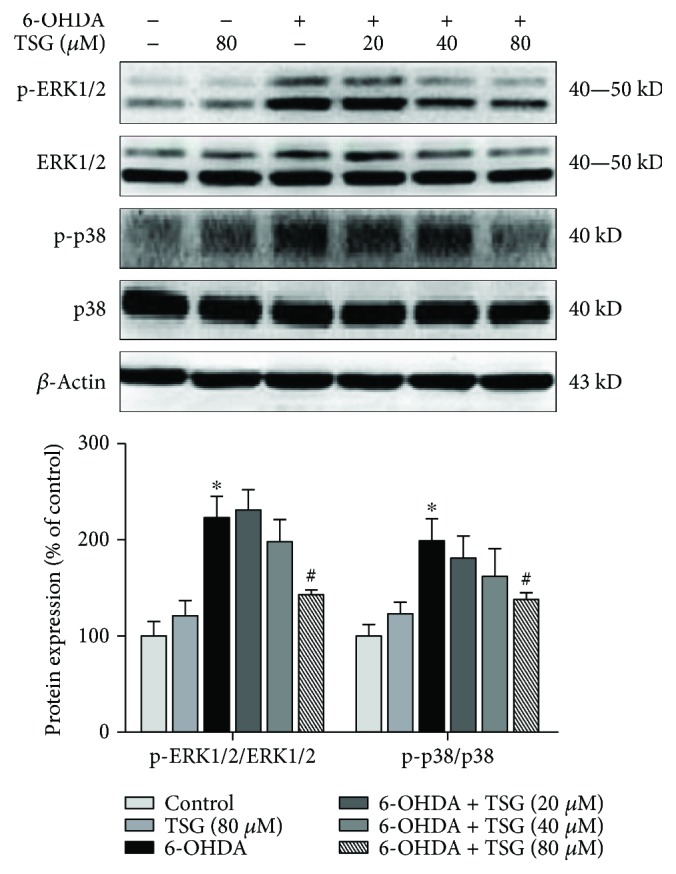
TSG attenuated 6-OHDA-induced MAPK pathway activation. The protein expressions of p-ERK1/2, ERK1/2, p-p38, and p38 were investigated by western blotting analysis. The ratio of densitometry values of p-ERK1/2 and p-p38 with total ERK1/2 and p38 was analyzed and normalized to each respective control group. Results were expressed as mean ± SEM from three independent experiments performed in triplicate. ^∗^*p* < 0.05 compared with the control groups; ^#^*p* < 0.05 compared with 6-OHDA-treated group.

It is well known that MAPK signaling pathway participated in the modulation of immune responses. Next, the effects of TSG on 6-OHDA-induced MAPK pathway activation were evaluated. Primary neuron-glia cocultures were treated with TSG for 30 min followed by the stimulation of 6-OHDA for 24 h. As shown in Figure 6, TSG attenuated 6-OHDA-induced ERK1/2 and p38 phosphorylation.

## 4. Discussion

This study demonstrated that TSG produced neuroprotection against 6-OHDA-induced DA neurotoxicity via the attenuation of microglia activation and the subsequent release of proinflammatory factors. These findings suggest that TSG might hold potential therapeutic effects on PD.

Recently, neuroinflammation has been confirmed to be involved in the etiology and pathogenesis of PD [14]. Microglia activation that participated in the modulation of neuroinflammation has been well documented [15]. Upon activation by brain injury and inflammogen stimuli, microglia excreted amounts of proinflammatory factors, such as cytokines and reactive oxygen species (ROS). The accumulation of these factors was recognized to lead to the progressive loss of DA neurons [16]. However, the dying/dead DA neurons also release various types of neurotoxic soluble factors such as *α*-synuclein and damage-associated molecular patterns (DAMPs), which in turn induced microglia reactivation. These activated microglia again released proinflammatory factors and caused the continuous DA neuronal damage [17]. Thus, a “self-propelling” vicious cycle was created to result in the gradually progressive DA neurodegeneration. 6-OHDA, a hydroxyl derivative of DA, competes for dopamine uptake sites and oxidized to neurotoxic substances upon injection into the SN of the brain. Neurotoxic action of 6-OHDA occurs through the accumulation of the toxin in DA neurons, followed by the alteration of cellular homeostasis and neuronal damage [18]. Till now, 6-OHDA-elicited DA neurodegeneration was a well-characterized experimental PD model both *in vivo* and *in vitro* [19]. In the present study, 6-OHDA induced DA neurons loss and then indirectly elicited microglia activation and consequent proinflammatory factor excretion. However, TSG seemed to produce DA neuroprotection via the inhibition of microglia activation and thus halt this vicious cycle running.

Next, to explore whether TSG was capable of reducing microglia-mediated neuroinflammatory response, the effects of TSG on NO, IL-1*β*, and TNF-*α* production were determined. Increasing evidence has demonstrated that the proinflammatory factors such as NO, IL-1*β*, and TNF*α* contributed to neurodegenerative diseases [20]. Postmortem data indicated that the extensive proliferation of reactive microglia and the elevated production of NO, IL-1*β*, and TNF-*α* were discerned in the SN region [21]. In addition, NO could react with ROS to form highly toxic intermediates such as peroxynitrite to induce DA neuronal damage [22]. Thus, inhibition of neuroinflammatory factor production might exhibit a promising therapeutic potential for PD. In line with our previous findings that TSG inhibited LPS-elicited proinflammatory factor production in BV2 cells [23], the present study demonstrated that TSG reduced 6-OHDA-elicited proinflammatory factor release. These findings suggested that amelioration of microglia activation and reduction of the subsequent proinflammatory factor generation were involved in TSG-mediated neuroprotection against 6-OHDA-induced DA neurotoxicity.

MAPK pathway is a well-known vital regulator of neuroinflammation [24]. Intensive studies showed that 6-OHDA-induced DA neuronal injury has been attributed to the activation of MAPK pathway [25]. Moreover, MAPK pathway regulates the release of proinflammatory cytokines from activated microglia including NO, IL-1*β*, and TNF-*α* [26]. Two members of the MAPK family, p38 and ERK1/2, were all activated upon neuroinflammation. Amounts of studies confirmed that ERK1/2 and p38 had been indicated to mediate neuroinflammatory reactions in AD and PD models [27, 28]. ERK1/2 can be activated by oxidative stress, inflammation, and cytokines [29], and a specific ERK1/2 inhibitor, U0126, conferred significant neuroprotection against DA neurodegeneration [30]. Besides, a series of studies have illustrated that the damaged DA neurons were accompanied by the increased number of p-p38 and iNOS-positive cells in the SN of PD mice model [31]. Therefore, the intervention of MAPK pathway activation is consequently applied for neurodegenerative disease treatment [32]. This study found that TSG inhibited 6-OHDA-elicited activation of MAPK pathway, implying that the inhibitory effects of TSG on proinflammatory factor production would likely result from the inactivation of MAPK pathway.

At present, current available drugs alleviate the symptoms of PD but are inadequate for halting the progression of PD. However, the adverse effects of the available drugs and nonmotor symptoms hold huge challenges for long-term therapy. Therefore, the new potential therapeutic agents and strategies are urgently required to delay or stop the progressive features of the disease [33]. Since neuroinflammation is considered to contribute to the acceleration of PD progression, this process might be an important breakpoint for the disease control. Thus, treatment with anti-inflammatory agents is gaining importance on the pharmacotherapy of PD. The present study revealed that TSG produced DA neuroprotection via its anti-inflammatory actions. However, further studies are required to fully illuminate the molecular mechanisms through which TSG potentiates anti-inflammatory activities.

## 5. Conclusion

This study here demonstrates that TSG produces neuroprotection from 6-OHDA-induced DA neurotoxicity through the inhibition of microglia-elicited neuroinflammation. Our data indicate that TSG might possess a beneficial potential for PD treatment.
